# Liposomes trigger bone marrow niche macrophage “foam” cell formation and affect hematopoiesis in mice

**DOI:** 10.1016/j.jlr.2022.100273

**Published:** 2022-09-07

**Authors:** Yue Li, Ran Yao, Miao Ren, Ke Yuan, Yuwei Du, Yuan He, Haiquan Kang, Shengnan Yuan, Wen Ju, Jianlin Qiao, Kailin Xu, Lingyu Zeng

**Affiliations:** 1 School of Medical Technology, Xuzhou Medical University, Xuzhou, Jiangsu, China; 2 Blood Diseases Institute, Xuzhou Medical University, Xuzhou, Jiangsu, China; 3 Key Laboratory of Bone Marrow Stem Cell, Xuzhou, Jiangsu, China; 4 Department of Hematology, The Affiliated Hospital of Xuzhou Medical University, Xuzhou, Jiangsu, China; 5 School of Pharmacy, Xuzhou Medical University, Xuzhou, Jiangsu, China; 6 Department of Laboratory Medicine, The Affiliated Hospital of Xuzhou Medical University, Xuzhou, Jiangsu, China

**Keywords:** liposome degradation, macrophage, lipid droplet, ER stress, sterile inflammation, side effect, nanocarrier, mononuclear phagocyte system, inflammatory cytokines, erythropoiesis, BM, bone marrow, DGAT, diacylglycerol acyltransferase, DiD, DiIC18(5) solid (1,1''-dioctadecyl-3,3, 3'',3''-tetramethylindodicarbocyanine, 4-chlorobenzenesulfonate salt), DiO, DiOC18(3) 3,3′-dioctadecyloxacarbocyanine perchlorate, ER, endoplasmic reticulum, HSC, hematopoietic stem cell, IF, immunofluorescence, IRE1α, inositol-requiring transmembrane kinase/endoribonuclease 1α, LD, lipid droplet, LT-HSC, long-term HSC, LSK, Lin^−^Sca1^+^c-Kit^+^, MPS, mononuclear phagocyte system, NP, nanoparticle, PFA, paraformaldehyde, RT-qPCR, quantitative real-time PCR, TAG, triacylglycerol

## Abstract

Liposomes are the most widely used nanocarrier platform for the delivery of therapeutic and diagnostic agents, and a number of liposomes have been approved for use in clinical practice. After systemic administration, most liposomes are cleared by macrophages in the mononuclear phagocyte system, such as the liver and bone marrow (BM). However, the majority of studies have focused on investigating the therapeutic results of liposomal drugs, and too few studies have evaluated the potential side effects of empty nanocarriers on the functions of macrophages in the mononuclear phagocyte system. Here, we evaluate the potential effects of empty liposomes on the functions of BM niche macrophages. Following liposome administration, we observed lipid droplet (LD) accumulation in cultured primary macrophages and BM niche macrophages. We found that these LD-accumulating macrophages, similar to foam cells, exhibited increased expression of inflammatory cytokines, such as IL-1β and IL-6. We further provided evidence that liposome deposition and degradation induced LD biogenesis on the endoplasmic reticulum membrane and subsequently disturbed endoplasmic reticulum homeostasis and activated the inositol-requiring transmembrane kinase/endoribonuclease 1α/NF-κB signaling pathway, which is responsible for the inflammatory activation of macrophages after liposome engulfment. Finally, we also showed the side effects of dysfunctional BM niche macrophages on hematopoiesis in mice, such as the promotion of myeloid-biased output and impairment of erythropoiesis. This study not only draws attention to the safety of liposomal drugs in clinical practice but also provides new directions for the design of lipid-based drug carriers in preclinical studies.

## Graphical abstract

**Figure:**
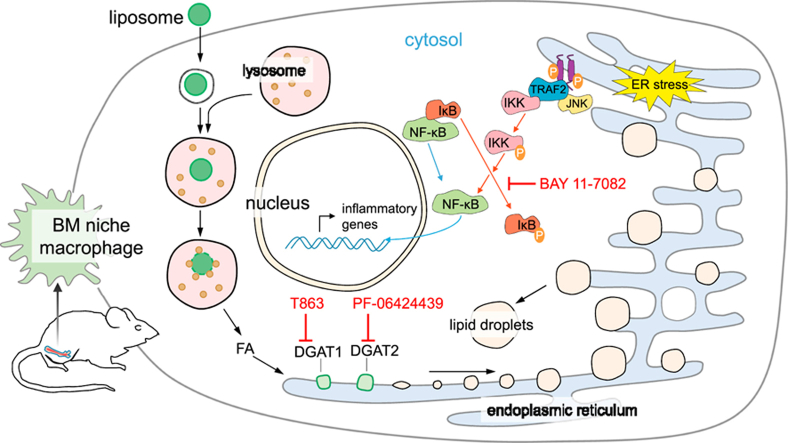

In recent decades, countless nanoparticles (NPs) have been designed for drug delivery, and a number of these NPs have been successfully applied in clinical practice (1). Lipid-based NPs are one of the NP platforms that are most commonly used for delivering small molecules, nucleic acids, peptides, and imaging dyes because these carriers are considered to be relatively safe drug carriers (2, 3, 4, 5). Among these carriers, liposomes are the most widely used and successfully commercialized lipid-based nanocarrier. Currently, there are over 20 liposomal products available on the market, such as Doxil, DepoCyt, and Marqibo (6).

Mostly, liposomes help to achieve controllable drug release or target-specific delivery. After decades of development, specificity of liposomal drug delivery systems in delivery drugs to target sites or cells has been significantly improved by optimizing the physicochemical properties and surface ligands of these systems (6, 7). However, after systemic administration, most liposomes are inevitably engulfed by resident macrophages and other phagocytic cells in the mononuclear phagocyte system (MPS), such as the liver, spleen, lungs, and bone marrow (BM) (8, 9, 10, 11). Are the functions of these phagocytic cells in the MPS affected after NP engulfment? Previous studies have reported the influence of liposomes on the function of Kupffer cells in the liver (12, 13, 14). However, none of the studies evaluated the effect of liposomes on the functions of macrophages in the BM.

Different from the liver and lungs, the BM is the primary site of new blood cell production (or hematopoiesis), and the BM is also termed the “fertile soil” that nurses the “seeds” hematopoietic stem cells (HSCs) (15, 16). Liposomes also accumulate in the BM after systemic administration (17, 18); should we consider the accumulation of NPs in the BM to be a microenvironmental contamination of the “fertile soil”? In the last 10 years, there have been substantial breakthroughs in the characterization of BM niche macrophages. BM niche macrophages are not only effector cells of the innate immune system but also involved in HSC maintenance and erythroid maturation at homeostasis (19, 20, 21, 22). Moreover, dysfunction of BM niche macrophages results in abnormal hematopoiesis, leading to various diseases (23, 24, 25, 26). Previous studies have shown that lipid-based NPs induce a long-term disruption in peripheral blood cells (27, 28). Do the changes in peripheral blood cells correlate with the dysfunction of BM niche macrophages after engulfing lipid-based NPs?

To bridge this knowledge gap, we studied the effects of liposome uptake on macrophage functions and BM hematopoiesis. We found that in vitro cultured macrophages acquire a proinflammatory phenotype after liposome uptake. These features are also observed in the BM niche macrophages of mice after systemic liposome administration. With the help of several inhibitors, we demonstrated that abnormal lipid droplet (LD) biogenesis induces endoplasmic reticulum (ER) stress and upregulates the expression of proinflammatory genes via the NF-κB signaling pathway. Finally, we investigated how dysfunctional BM niche macrophages affect hematopoiesis after liposome administration. Together, these findings indicate that liposomes induce proinflammatory activation of BM niche macrophages and further affect their ability to regulate hematopoiesis. This study not only draws attention to the safety of nanomedicines in clinical practice but also provides new approaches for the design of lipid-based drug carriers for use in preclinical studies.

## Materials and methods

### Animals

Male C57BL/6 mice were obtained from Beijing Vital River Laboratory Animal Technology Co Ltd. The mice were 6–8 weeks old when they were used in these experiments. The Animal Research Ethics Committee of Xuzhou Medical University approved all the experimental procedures.

### Primary cell culture and stimulation

A single-cell suspension of BM was prepared by flushing the BM cavity. Then mouse BM cells were cultured in complete RPMI-1640 medium containing 10% denatured FBS, penicillin-streptomycin (100 U/ml), and 50 ng/ml recombinant mouse macrophage colony-stimulating factor (catalog no.: 576404; BioLegend) for 6 days, and supplemented with macrophage colony-stimulating factor on day 3 by changing fresh medium. On day 6, macrophages were harvested for further identification and experiments.

For in vitro stimulation, macrophages were maintained in complete RPMI-1640 medium supplemented with 100 ng/ml lipopolysaccharide (catalog no.: L4391; Sigma-Aldrich), 50 ng/ml IFN-γ (catalog no.: 575302; BioLegend), or 50 ng/ml IL-4 (catalog no.: 574304; BioLegend) for 24 h. In some experiments, macrophages were treated with 20 μM T863 and 20 μM PF-06424439 or 10 μM BAY 11-7085. The indicated inhibitors were purchased from MedChemExpress.

### Flow cytometry

Cells were stained with the indicated antibodies for 30 min in ice-cold fluorescence-activated cell sorting buffer (PBS supplemented with 2% FBS). The following antibodies were used: APC anti-mouse F4/80 (BM8; BioLegend), FITC anti-mouse F4/80 (BM8; BioLegend), BV421 anti-mouse CD11b (M1/70; BioLegend), APC anti-mouse CD86 (GL-1; BioLegend), PE/Cyanine7 anti-mouse CD206 (C068C2; BioLegend), PE anti-mouse CD71 (RI7217; BioLegend), FITC anti-mouse Ter119 (TER-119; BD), FITC anti-mouse CD11b (M1/70; BioLegend), PE anti-mouse Gr-1 (RB6-8C5; BD), PE-Cy7 anti-mouse CD150 (TC15-12F12.2; BD), BV421 anti-mouse CD48 (HM48-1; BioLegend), PE anti-mouse c-Kit (ACK2; BD), and APC anti-mouse Sca-1 (W18174A; BD). For LD quantitative analysis, the cells were incubated in RPMI-1640 medium supplemented with 1 μg/ml BODIPY 493/503 for 30 min in 37°C incubator. Then, cells were washed and used for flow cytometry. Doublets and dead cells were excluded on the basis of forward scatter and side scatter distribution and 7-AAD (BioLegend) exclusion. The data were acquired by flow cytometry on an LSRFortessa™ cell analyzer (BD Biosciences) and analyzed with FlowJo, version 10.1 (Tree Star).

### Preparation of liposomes

Liposomes were prepared by the ethanol injection method as described before (29). Briefly, 10 mg phospholipids (l-α-phosphatidylcholine from soybean; CAS no.: 8002-43-5; Aladdin) and 5 mg cholesterol (CAS no.: 57-88-5; Aladdin) were dissolved in 500 μl ethanol. The resulting organic phase was added dropwise to 4 ml PBS with 100 rpm/min stirring. Then, the organic solvent was removed by ultracentrifugation of the liposome suspension at 14,000 rpm for 1 h. The obtained precipitates were dispersed in PBS and stored at 4°C. Endotoxin contamination in the stocked liposome solution was evaluated by the limulus amoebocyte lysate chromogenic method. The endotoxin levels of two representative batches of prepared liposomes were 0.0139 and 0.0121 endotoxin units/ml, respectively. After dilution with water, the particle size and zeta potential of the liposomes were measured by a Zetasizer Nano-ZS (Malvern Instruments, UK). For DiD (DiIC18(5) solid [1,1''-dioctadecyl-3,3, 3'',3''-tetramethylindodicarbocyanine, 4-chlorobenzenesulfonate salt])-loaded liposomes or DiO (DiOC18(3) 3,3′-dioctadecyloxacarbocyanine perchlorate)-loaded liposomes, the DiD (or DiO) stock solution was added to the above ethanolic solution.

### RNA extraction and quantitative PCR

Total RNA was extracted from macrophages and purified using TRIzol™ Reagent (catalog no.: 15596026; Invitrogen). Reverse transcription of RNA transcripts into complementary DNA was performed with the PrimeScript™ RT Reagent Kit (catalog no.: RR037A; TaKaRa). Quantitative real-time PCR (RT-qPCR) assays were performed. Transcription levels were determined with 2X Universal SYBR Green Fast qPCR Mix (catalog no.: RK21203; ABclonal) using the LightCycler® 480 System (Roche). The expression levels of the target genes were normalized to that of the housekeeping gene *β-actin*. In addition, the relative gene expression of the treated group was normalized to that of the control group to obtain the relative fold change in expression.

All the primers were synthesized by GENEWIZ, and their sequences were as follows: *β-actin*, 5′-GTGACGTTGACATCCGTAAAGA-3′ (forward) and 5′-GCCGGACTCATCGTACTCC-3′ (reverse); *CD86*, 5′-TCAATGGGACTGCATATCTGCC-3′ (forward) and 5′-GCCAAAATACTACCAGCTCACT-3′ (reverse); *CD206*, 5′-CTCTGTTCAGCTATTGGACGC-3′ (forward) and 5′-TGGCACTCCCAAACATAATTTGA-3′ (reverse); *Arg1*, 5′-CATTGGCTTGCGAGACGTAGAC-3′ (forward) and 5′-GCTGAAGGTCTCTTCCATCACC-3′ (reverse); *NOS2*, 5′-GAGACAGGGAAGTCTGAAGCAC-3′ (forward) and 5′-CCAGCAGTAGTTGCTCCTCTTC-3′ (reverse); *IL-6*, 5′-TACCACTTCACAAGTCGGAGGC-3′ (forward) and 5′-CTGCAAGTGCATCATCGTTGTTC-3′ (reverse); *IL-1β*, 5′-GAAATGCCACCTTTTGACAGTG-3′ (forward) and 5′-TGGATGCTCTCATCAGGACAG-3′ (reverse); *DGAT1*, 5′-GGAATATCCCCGTGCACAA-3′ (forward) and 5′-CATTTGCTGCTGCCATGTC-3′ (reverse); *DGAT2*, 5′-*CTGTGCTCTACTTCACCTGGCT-3′* (forward) and 5′-CTGGATGGGAAAGTAGTCTCGG-3′ (reverse); *CHOP*, 5′-GGAGGTCCTGTCCTCAGATGAA-3′ (forward) and 5′-GCTCCTCTGTCAGCCAAGCTAG-3′ (reverse); *ATF4*, 5′-AACCTCATGGGTTCTCCAGCGA-3′ (forward) and 5′-CTCCAACATCCAATCTGTCCCG-3′ (reverse); *Bip*, 5′-TCAGCATCAAGCAAGGATTG-3′ (forward) and 5′-AAGCCGTGGAGAAGATCTGA-3′ (reverse); *XBP1*, 5′-TGGACTCTGACACTGTTGCCTC-3′ (forward) and 5′-TAGACCTCTGGGAGTTCCTCCA-3′ (reverse); and *spl-XBP1*, 5′-GAGTCCGCAGCAGGTG-3′ (forward) and 5′-GTGTCAGAGTCCATGGGA-3′ (reverse).

### In vivo animal experiments

To evaluate the effect of liposomes on BM niche macrophages in vivo, liposomes were intravenously injected into mice. The lipid dosage of the injected liposomes was 200 mg/kg (approximately 4 mg/mouse). The bones and peripheral blood samples were harvested for analysis at the indicated time points. The antibody treatments were administered via intraperitoneal injection at the indicated time points after liposome injection. Anti-IL-6 (MP5-20F3; BioLegend) and anti-IL-1β (B122; BioLegend) antibodies or the respective controls were used at doses of 10 mg/kg.

### Quantification of cytokine levels in BM extracellular fluid

To collect BM extracellular fluids for analysis, one femur was dissected at the indicated time points after liposome injection and immediately flushed into 300 μl ice-cold PBS. The solution was centrifuged at 15,000 *g* for 5 min. The supernatant was stored at −80°C. Cytokine levels were measured with ELISA kits (BioLegend) according to the manufacturer’s instructions.

### Organelle staining in live cells

Macrophages were seeded on confocal dishes (Nest). For LD staining, macrophages were incubated in medium supplemented with 1 μg/ml BODIPY 493/503 (Invitrogen) for 30 min. For lysosome staining, macrophages were incubated in medium supplemented with 100 nM LysoTracker Red (Invitrogen) for 30 min. For ER staining, macrophages were first washed twice with HBSS (supplemented with Ca^2+^ and Mg^2+^) and then were incubated in diluent supplemented with 500 nM ER-Tracker Red (Beyotime) for 30 min. The nucleus was stained with 2 μg/ml Hoechst 33342 for an additional 2 min. After incubation, the cells were washed twice for live-cell imaging.

### Immunocytochemical staining

To examine LD accumulation in BM macrophages, single cells from the BM cavity were incubated in complete medium supplemented with 1 μg/ml BODIPY 493/503 for 3 h and then with BV421 anti-mouse CD11b antibody (M1/70; BioLegend) and APC anti-mouse F4/80 antibody (BM8; BioLegend) for an additional 30 min at room temperature. To stain perilipin 2 of LDs, macrophages were freshly fixed with 4% paraformaldehyde (PFA) for 10 min, and the cell membrane was permeabilized with 0.01% digitonin for 30 min. Anti-perilipin 2 antibodies were incubated overnight at 4°C, followed by the secondary antibodies for 2 h at room temperature, 1 μg/ml BODIPY 493/503 (Invitrogen) for 30 min, and Hoechst 33342 for 30 min at room temperature. After incubation, the cells were washed twice and used for imaging.

### Cryosectioning and immunofluorescence staining

Bones were freshly dissected and fixed in ice-cold 4% PFA for 6 h at 4°C. Then, the bone samples were washed with PBS under gentle agitation overnight at 4°C to remove PFA. After dehydration with 30% sucrose for at least 2 days, the bones were embedded in OCT and bisected by a Leica cryostat (Leica, Germany). Half-bones were washed with PBS to remove OCT and blocked with 2% BSA for 30 min. Primary antibodies were incubated overnight at 4°C, followed by the secondary antibodies for 2 h at room temperature, and Hoechst 33342 for 30 min at room temperature. After incubation, half-bones were washed twice with PBS for imaging. The following antibodies were used: rat anti-mouse endomucin monoclonal antibody (eBioV.7C7; Invitrogen) and FITC anti-rat secondary antibody.

### Confocal imaging

Images were acquired using a Zeiss LSM 880 fluorescence confocal microscope (Zeiss, Germany) with fixed lasers (405, 488, 561, and 633 nm). We acquired BODIPY493/503, DiO, or FITC images at 488 nm excitation, Hoechst 33342 or BV421 images at 405 nm excitation, and APC or DiD images at 633 nm excitation. Images were subsequently imported into ImageJ software (National Institutes of Health) for analysis.

### In vivo biodistribution of liposomes

To map the in vivo biodistribution of liposomes, DiD-loaded liposomes (25 μg DiD per mouse) were intravenously injected into the mice. On day 1 after liposome injection, the bones were harvested for fluorescence imaging by the IVIS® Lumina S5™ (PerkinElmer).

### Peripheral blood analyses and BM cell number counting

Peripheral blood was collected in tubes containing EDTA. Blood cell counts were obtained with a hematology analyzer (Mindray, China). To count BM cells, the BM cells were processed to generate single-cell suspensions. Total BM cell numbers were determined using a hematology analyzer (Mindray). The number of different cell types in the BM was calculated based on BM cell counting and flow cytometry analysis with appropriate markers.

### Statistical analysis

The data were analyzed with GraphPad Prism software (version 8.0; GraphPad Software, Inc). Comparisons for two groups were calculated using unpaired two-tailed Student’s *t*-tests, comparisons of more than two groups were calculated using one-way ANOVA with multiple comparison tests. The data were tested for normal distribution before analysis. The data are presented as the mean ± SD. Differences were considered significant when *P* values were below 0.05.

## Results

### BM niche macrophages engulf liposomes after systemic administration

**Fig. 1:**
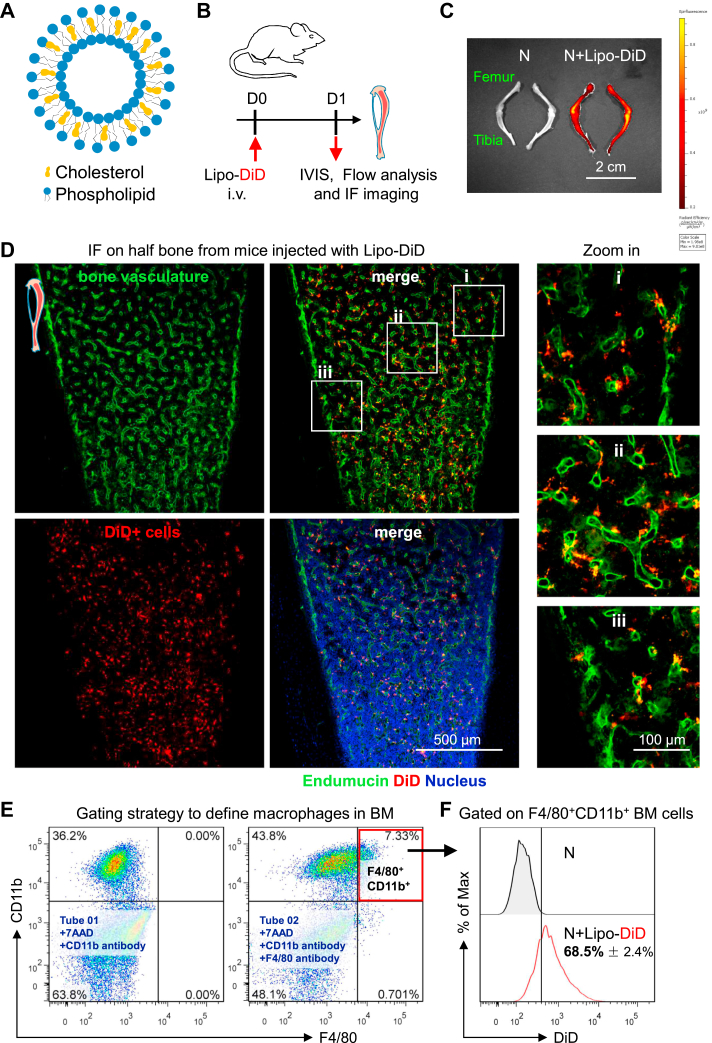
Liposomes accumulate in BM niche macrophages after systemic administration. A: Schematic structure of liposomes. B: Experimental setup. Liposomes were intravenously injected into the tail veins of mice. On day 1 after liposome injection, bones were harvested for analysis. C: Representative in vivo imaging system images of bones from normal mice (N) and normal mice injected with DiD-loaded liposomes (N + Lipo-DiD). D: IF was used to investigate the distribution of DiD-loaded liposomes in bones after systemic injection; Endomucin (green color), DiD (red color), nuclei (blue color, Hoechst 33342); the scale bar represents 500 μm. E: Gating strategy for the quantification of BM niche macrophages. F: A flow cytometer was used to assess the DiD fluorescence intensity of F4/80^+^CD11b^+^ bone marrow cells in mice after DiD-loaded liposome injection. The data were calculated from individual mice (n = 3). All experiments were repeated at least twice, and representative results are shown.

To evaluate the potential side effects of liposomes, we prepared liposomes by the ethanol injection method in this study (29). The main components of liposomes are natural phospholipids and cholesterol (Fig. 1A). Physicochemical properties, including size, charge, and lipid composition, have been shown to affect the efficiency of cell targeting (30). Most clinical materials are medium sized (∼10–300 nm in diameter) (5, 7). In this study, the mean diameter of the liposomes was approximately 160 nm (supplemental Fig. S1A), and the mean zeta potential was approximately −5.9 mV when the liposomes were dissolved in water (supplemental Fig. S1B).

To evaluate the biodistribution of liposomes in vivo, the liposomes were labeled with the lipophilic carbocyanine dye (DiD) (Fig. 1B). Using an in vivo imaging system, strong DiD fluorescence signals were detected in the bones of mice on the first day after systemic injection (Fig. 1C). These results suggested that DiD-loaded liposomes were engulfed by BM cells. Next, we investigated the distribution of liposomes in the BM at single-cell resolution. The BM is a highly vascularized tissue in which the BM vasculature works in coordination with other perivascular stromal cells to support and regulate hematopoiesis (15, 16). Immunofluorescence imaging showed that many perivascular cells were labeled with the DiD dye (Fig. 1D). Flow cytometry was used to determine the identity of these DiD-positive cells. We found that most DiD-positive cells were positive for macrophage markers (CD11b and F4/80) (supplemental Fig. S1C). Moreover, approximately 70% of CD11b- and F4/80-positive BM niche macrophages were stained with the DiD dye (Fig. 1E, F). These results indicated that a high proportion of BM niche macrophages engulfed the liposomes after systemic administration. BM niche macrophages play a pivotal role in the regulation of BM hematopoiesis; however, the effect of liposome engulfment on BM niche macrophage functions has not been clearly elucidated.

### Liposome uptake induces proinflammatory activation of macrophages in vitro and in vivo

To investigate the effect of liposome uptake on macrophage functions, we used mouse BM cells to develop primary macrophages (supplemental Fig. S2A, B). Herein, we developed two subtypes of activated (proinflammatory M1 and anti-inflammatory M2) macrophages from naive (M0) macrophages using different cytokines (31) (supplemental Fig. S2A). The RT-qPCR results showed that M1 macrophages featured high levels of *CD86* and *NOS2* expression, whereas M2 macrophages have elevated expression of *CD206* and *Arginase-1* (supplemental Fig. S2C), indicating that naive macrophages had been successfully polarized into the activation states as expected.

**Fig. 2:**
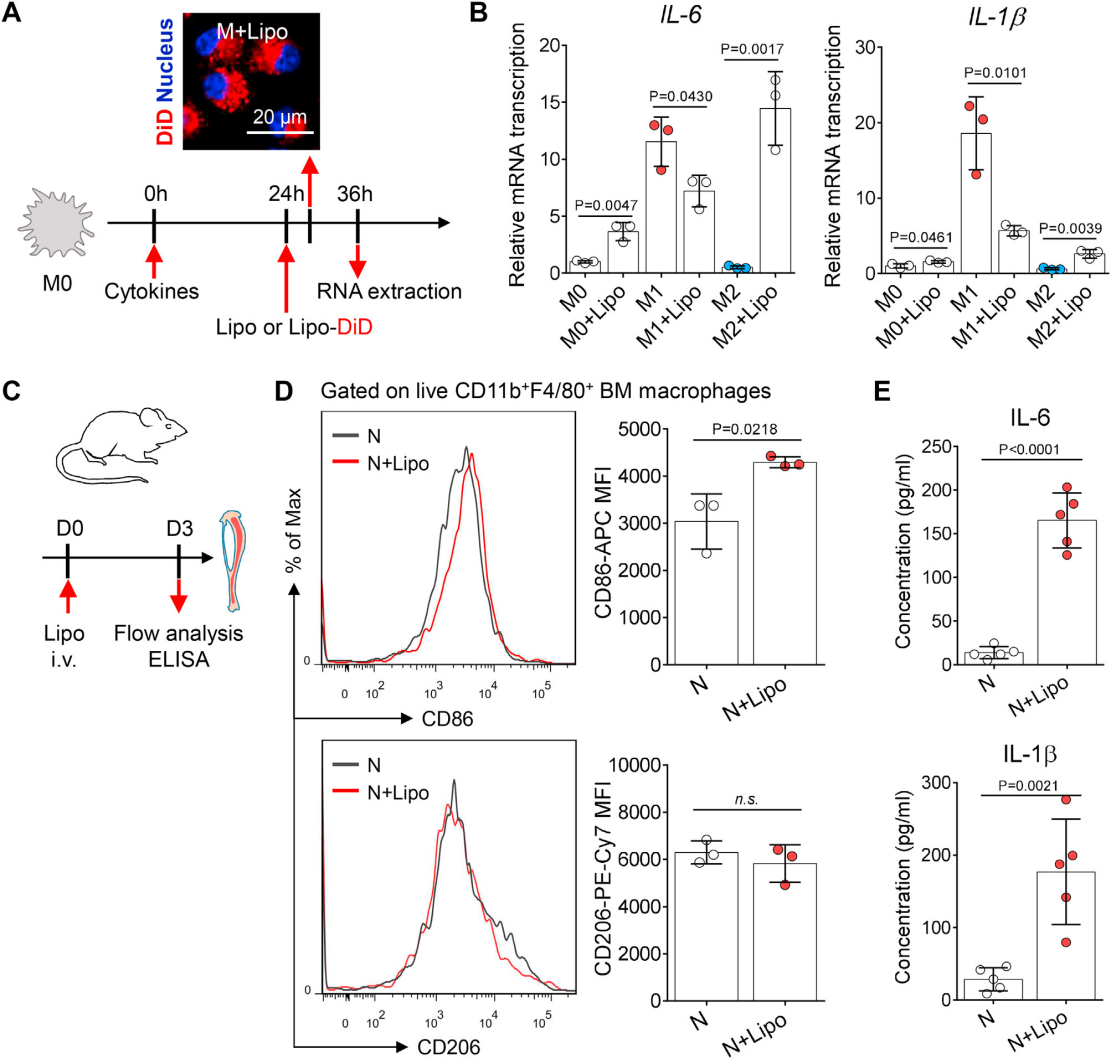
Liposome administration induces the inflammatory activation of macrophages. A: Schematic overview of experimental procedures. Representative fluorescence images of liposome deposition (red color, DiD) in macrophages (blue color, nucleus). The lipid dosage of liposomes for in vitro cell treatment was 1 mg/ml. The scale bar represents 20 μm. B: Twelve hours after liposome administration, RT-qPCR was used to assess the *IL-6* and *IL-1β* gene expression in activated macrophages. One dot represents the data from macrophages derived from one mouse (n = 3). C: Experimental setup. On day 3 after intravenous liposome injection, bones were harvested for analysis. D: Flow cytometry was used to assess the surface marker (CD86 and CD206) expression of F4/80^+^CD11b^+^ BM macrophages from normal mice (N) and normal mice injected with liposomes (N + Lipo). CD86 and CD206 fluorescence (MFI) in untreated BM niche macrophages or those treated with liposomes. The dots represent data from individual mice (n = 3). E: Levels of the indicated inflammatory cytokines in the extracellular fluid of the BM of mice after liposome administration. The dots represent data from individual mice (n = 5). All experiments were repeated at least twice, and representative results are shown. MFI, mean fluorescence intensity.

Next, we administered liposomes to activated macrophages in vitro. Using confocal microscopy, we found that macrophages engulfed DiD-loaded liposomes 2 h after administration (Fig. 2A). The RT-qPCR results showed that liposome-treated M2 macrophages had increased gene expression of M1 markers (such as *CD86* and *NOS2*) and decreased gene expression of M2 markers (such as *CD206* and *Arginase-1*) (supplemental Fig. S2D). These findings indicated that liposome uptake elicited the repolarization of M2 macrophages toward proinflammatory phenotype. Tanaka *et al.* (32) reported similar findings, namely, that liposomes upregulate the expression of CD80 and CD86 in BM-derived dendritic cells. Moreover, liposome uptake elevated the gene expression of proinflammatory cytokines (*IL-6* and *IL-1β*) in M2 macrophages (Fig. 2B).

To investigate whether the effect of liposome uptake on macrophage activation is still significant in situ, we also analyzed the changes in surface marker expression in BM macrophages before/after liposome administration (Fig. 2C). The flow cytometry results showed that liposome administration upregulated the protein expression of CD86 in BM niche macrophages (Fig. 2D). The changes in the expression of the surface marker CD206 in BM niche macrophages were not significant (Fig. 2D), which might occur because of the retention of protein markers (33). We also analyzed the levels of proinflammatory cytokines in the extracellular fluid of the BM of liposome-treated mice. The ELISA results showed that the levels of IL-6 and IL-1β in the BM extracellular fluid were significantly increased after liposome administration (Fig. 2E). We demonstrated that after extravasation, most liposomes were engulfed by BM niche macrophages (supplemental Fig. S1C). Therefore, the increased proinflammatory cytokines in the extracellular fluid of the BM might result from the proinflammatory activation of BM niche macrophages after liposome uptake. Overall, these findings suggest that liposome uptake induces proinflammatory activation of macrophages in vitro and in vivo. However, further experiments are needed to investigate how liposome uptake supports the proinflammatory activation of M2 macrophages.

### Liposome-treated macrophages exhibit LD accumulation in vitro and in vivo

Foam cells are fat-laden M2 macrophages, and they can secrete proinflammatory cytokines to maintain the local inflammatory microenvironment at the sites of lesions (34, 35, 36). Inspired by the formation of foam cells, we speculate that liposome degradation and its cascades might correlate with the inflammatory activation of M2 macrophages after liposome administration.

**Fig. 3:**
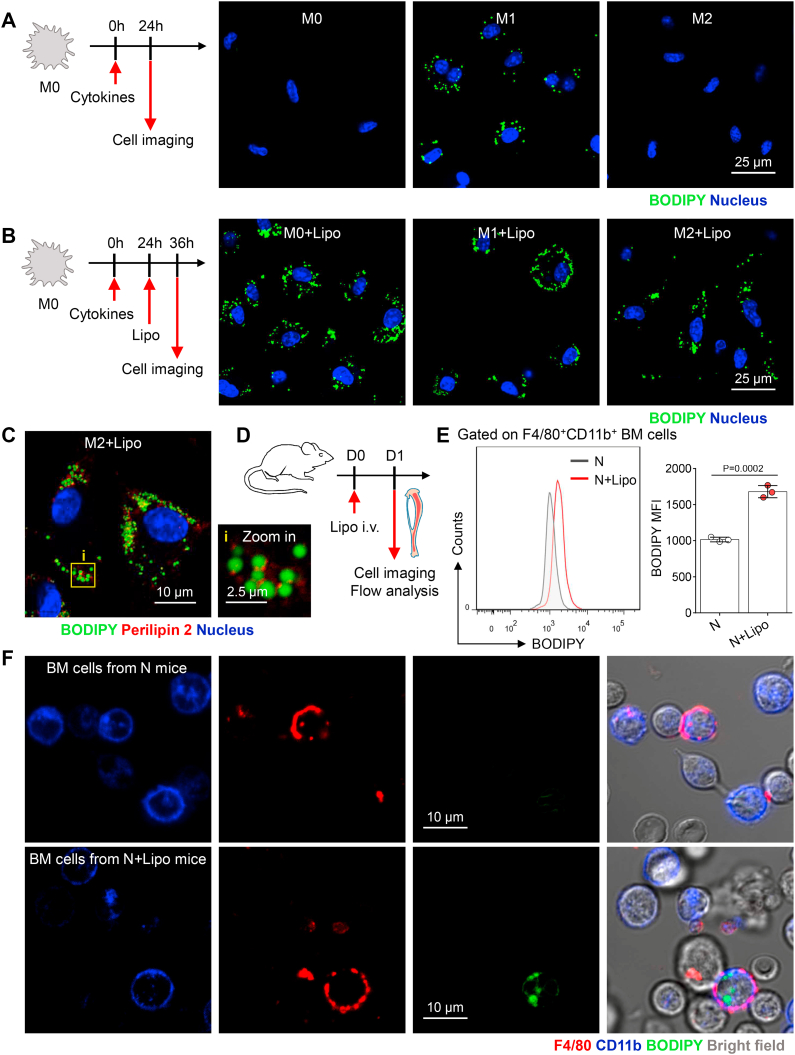
Liposome administration induces LD accumulation in macrophages. A: Experimental setup. Representative fluorescence confocal microscopy images of LDs (green color, BODIPY) and nuclei (blue color, Hoechst 33342) in macrophages before liposome administration. The scale bar represents 25 μm. B: Experimental setup. Representative fluorescence confocal microscopy images of LDs (green color, BODIPY) and nuclei (blue color, Hoechst 33342) in macrophages after liposome administration. The scale bar represents 25 μm. C: Representative fluorescence confocal microscopy images of LDs (green color, BODIPY), perilipin 2 (red color), and nuclei (blue color, Hoechst 33342) in macrophages after liposome administration. The scale bar represents 10 μm. D: Experimental setup. On day 1 after intravenous liposome injection, bones were harvested for analysis. E: Flow cytometry was used to assess the BODIPY fluorescence intensity of F4/80^+^CD11b^+^ BM macrophages from normal mice (N) and normal mice injected with liposomes (N + Lipo). Bodipy 493/503 fluorescence (MFI) in untreated BM niche macrophages or those treated with liposomes. The dots represent data from individual mice (n = 3). F: Representative fluorescence images of LDs in BM niche macrophages after immunocytochemistry (ICC) and free BODIPY staining. Cell morphology (gray color, bright field), LDs (green color, BODIPY), F4/80 (red color), and CD11b (blue color). The scale bar represents 10 μm. All data shown were representative of two independent runs of experiments. All experiments were repeated at least twice, and representative results are shown. MFI, mean fluorescence intensity.

Similar to the compositions of LDLs, a liposome is a spherical vesicle that contains phospholipids and cholesterol molecules. Previously, we found that unlike nonlipid NPs, liposomes could induce LD formation in macrophages. In this study, BODIPY dye was used to stain LDs in macrophages before/after liposome administration. Using confocal microscopy, we found that M1 macrophages exhibited greater LD accumulation than resting M0 and activated M2 macrophages before liposome administration (Fig. 3A), which was consistent with previous reports (37, 38). The administration of liposomes induced BODIPY-positive droplet accumulation in M0 and M2 macrophages, producing foam cell-like macrophages (Fig. 3B). Perilipin 2 (also known as adipophilin) is an LD-associated protein and exclusively localized to the surface of LDs (39). Immunofluorescence imaging showed that the BODIPY-positive droplets in liposome-treated M2 macrophages were also positive for perilipin 2, confirming their LD identity (Fig. 3C). Moreover, we found that another commercial liposome (containing phosphatidylcholine and cholesterol, provided by Liposoma BV) also induced LD formation and sterile inflammation in M2 macrophages (supplemental Fig. S3A, B). This is consistent with previous studies showing that various lipid-based NPs that contain different phospholipids efficiently induce LD accumulation in macrophages (40, 41).

To investigate whether LDs accumulate in BM niche macrophages after systemic liposome administration, mouse BM was harvested to prepare a single-cell suspension on day 1 after administration; these cells were stained with BODIPY dye for analysis (Fig. 3D). The flow cytometry results showed that the F4/80^+^CD11b^+^ BM macrophages from liposome-injected mice had a higher BODIPY fluorescence intensity than those from normal mice (Fig. 3E). Morphologically, using confocal microscopy, we found that F4/80^+^CD11b^+^ BM macrophages accumulated LDs after liposome injection (Fig. 3F). Collectively, these data suggest that liposome uptake triggers LD biogenesis in macrophages in vitro and in vivo, but whether this contributes to the reactivation of macrophages remains unknown.

### LD biogenesis contributes to macrophage inflammation after liposome engulfment

**Fig. 4:**
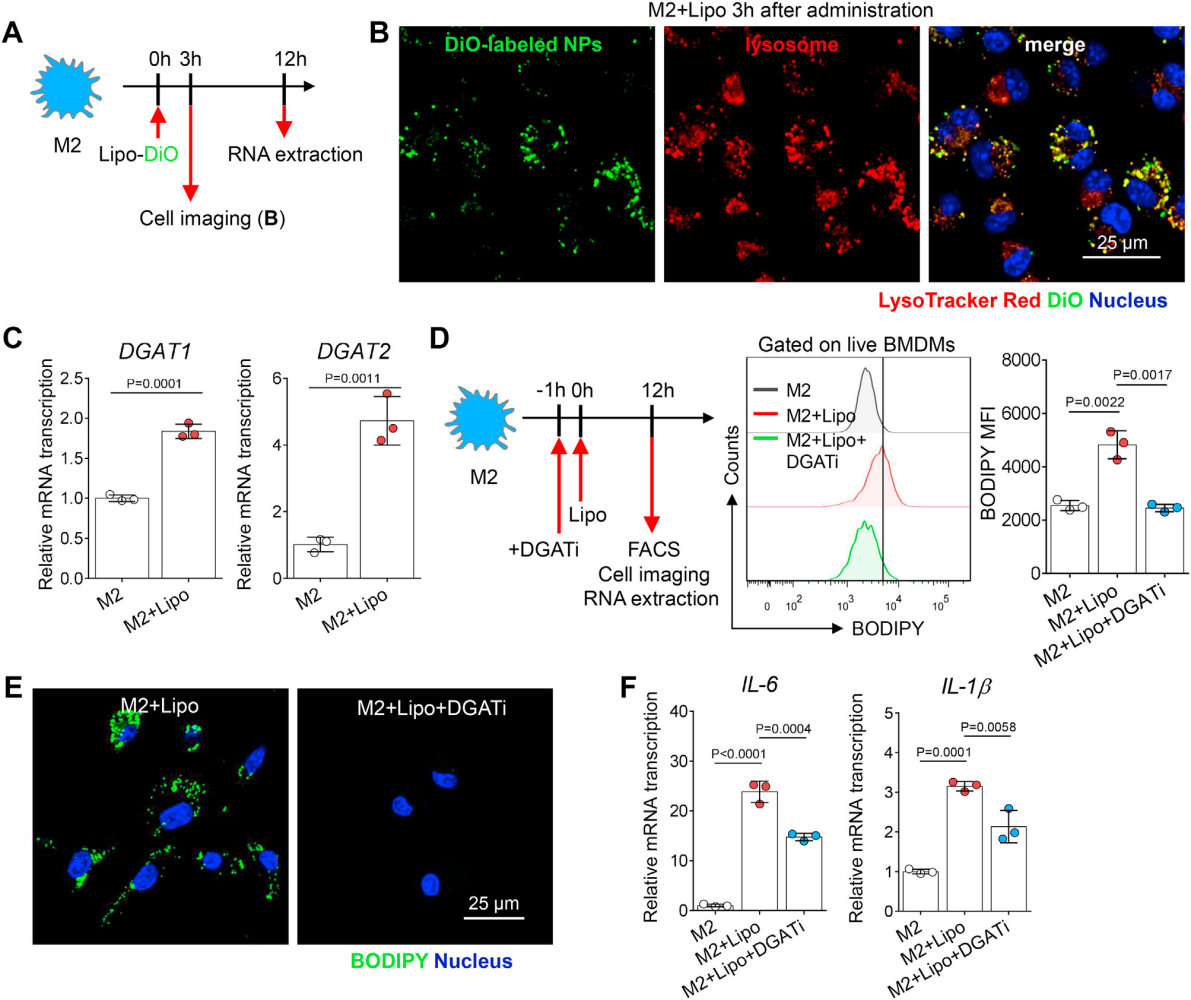
LD formation correlates with the inflammatory activation of macrophages. A: Experimental setup. DiO-loaded liposomes were administered to in vitro cultured macrophages; 3 h after liposome administration, macrophages were counterstained with LysoTracker Red and Hoechst 33342. B: Fluorescence confocal images of labeled liposomes and lysosomes in macrophages; liposomes (green color, DiO), lysosomes (red color, LysoTracker), and nuclei (blue color, Hoechst 33342); the scale bar represents 25 μm. C: Twelve hours after liposome administration, RT-qPCR was used to assess *DGAT1* and *DGAT2* gene expression of M2 macrophages. One dot represents the data from macrophages derived from one mouse (n = 3). D: Experimental setup. Macrophages were analyzed 12 h after liposome administration in the presence or the absence of DGAT inhibitors. Then, flow cytometry was used to assess the BODIPY fluorescence intensity of M2 macrophages after treatment (M2, M2 + Lipo, M2 + Lipo + DGATi). BODIPY 493/503 fluorescence (MFI) in untreated macrophages or those treated with liposomes and inhibitors. One dot represents the data from macrophages derived from one mouse (n = 3). E: Representative fluorescence confocal microscopy images of LDs (green color, BODIPY) and nuclei (blue color, Hoechst 33342) in M2 macrophages after treatment (M2 + lipo, M2 + lipo + DGATi). The scale bar represents 25 μm. F: RT-qPCR was used to assess the *IL-6* and *IL-1β* gene expression of M2 macrophages after treatment (M2, M2 + Lipo, M2 + Lipo + DGATi). One dot represents the data from macrophages derived from one mouse (n = 3). All experiments were repeated at least twice, and representative results are shown. MFI, mean fluorescence intensity.

LDs are increasingly recognized as dynamic players in immune regulation (42, 43). Recent studies have demonstrated that macrophages enhance LD biogenesis to support host defense when exposed to inflammatory stimuli, such as parasites, bacteria, and viruses (44, 45, 46). To elucidate the relationship between liposome degradation and LD formation in macrophages, LysoTracker Red dye was used to stain lysosomes in M2 macrophages after DiO-loaded liposome administration (Fig. 4A). Using confocal microscopy, we found that DiO-labeled vesicles overlapped with LysoTracker Red-positive lysosomes (Fig. 4B), indicating that liposomes accumulated in lysosomes after endocytosis. This finding is consistent with a previous report that liposomes are ingested by macrophages via endocytosis, and endosomes subsequently fuse with lysosomes containing phospholipases (47). Lysosomal phospholipases can disrupt the bilayers of liposomes and hydrolyze phospholipids to produce free fatty acids (48), providing the common ingredients for LD formation in macrophages.

Triacylglycerols (TAGs) are the major components of LDs, and acyl-CoA:diacylglycerol acyltransferase (DGAT) enzymes are the only enzymes committed to TAG biosynthesis (49). We also investigated the relative gene expression of enzymes responsible for LD biogenesis. Using RT-qPCR, we found that the gene expression of *DGAT-1* and *DGAT-2* was elevated in liposome-treated macrophages, indicating that these macrophages had enhanced LD biogenesis capabilities (Fig. 4C). Castoldi *et al.* (38) found that the inhibition of TAG synthesis suppresses the production of inflammatory cytokines, such as IL-1β and IL-6. To determine the role of TAG synthesis in the production of proinflammatory cytokines, we used a DGAT1 inhibitor (T863) and a DGAT2 inhibitor (PF-06424439) to pretreat macrophages under liposome administration (Fig. 4D). The confocal microscopy and flow cytometry results showed that the DGAT inhibitors suppressed LD development and accumulation (Fig. 4D, E) and the expression of proinflammatory genes in macrophages treated with liposomes (Fig. 4F). Taken together, the data indicate that the development of LDs supports the proinflammatory activation of M2 macrophages after liposome uptake.

### Liposome deposition-induced LD biogenesis initiates macrophage ER stress and upregulates proinflammatory gene expression

**Fig. 5:**
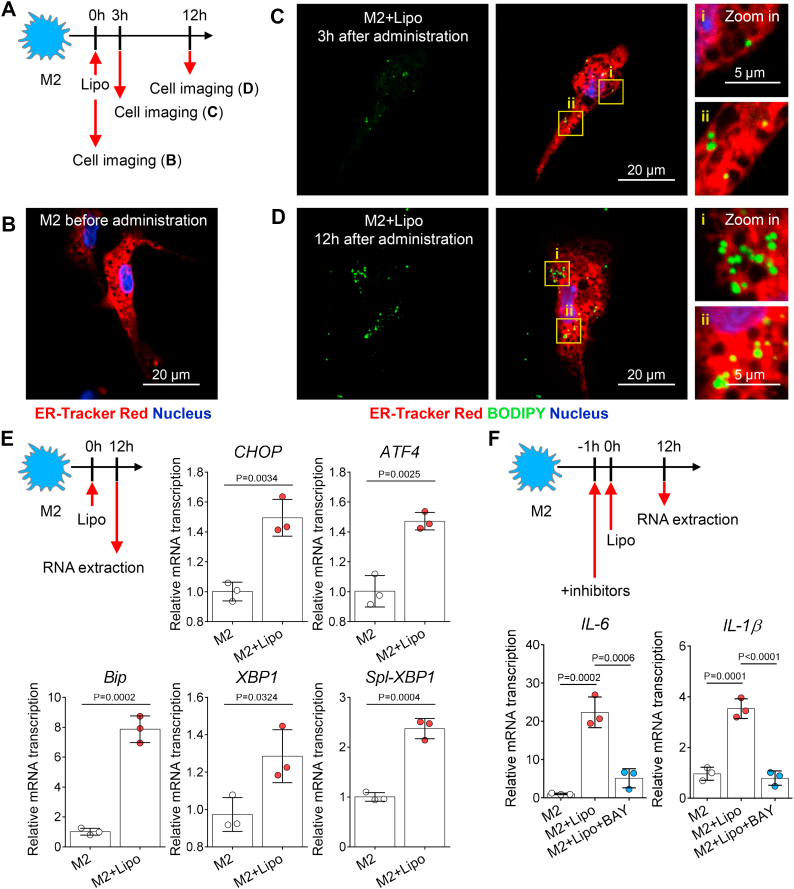
Liposome-induced LD biogenesis disrupts ER homeostasis in macrophages. A: Experimental setup. Liposomes were administered to in vitro-cultured macrophages; 3 h and 12 h after liposome administration, macrophages were counterstained with BODIPY, ER-Tracker Red, and Hoechst 33342. B: Representative fluorescence confocal microscopy images of ER (red color, ER-Tracker Red) and nuclei (blue color, Hoechst 33342) in macrophages before liposome administration. The scale bar represents 20 μm. C, D: Representative fluorescence confocal microscopy images of LDs (green color, BODIPY), ER (red color, ER-Tracker Red), and nuclei (blue color, Hoechst 33342) in macrophages 3 h or 12 h after liposome treatment. The scale bar represents 20 μm. E: Experimental setup. RT-qPCR was used to assess the ER stress-related gene (*CHOP*, *ATF4*, *Bip*, *XBP1*, and *Spl-XBP1*) expression in M2 macrophages after treatment (M2, M2 + Lipo). One dot represents the data from macrophages derived from one mouse (n = 3). F: RT-qPCR was used to assess the *IL-6* and *IL-1β* gene expression of M2 macrophages after 12 h of liposome treatment in the presence or the absence of NF-κB inhibitors (M2, M2 + Lipo, M2 + Lipo + BAY). One dot represents the data from macrophages derived from one mouse (n = 3). All experiments were repeated at least twice, and representative results are shown.

LD formation occurs on the cytoplasmic side of the ER membrane (50). The ER supplies most molecules required for LD biogenesis (49). In this study, ER-Tracker dye was used to stain the ER in M2 macrophages before/after liposome administration (Fig. 5A, B). Using confocal microscopy, we found that a few small LDs overlapped with the ER tubules 3 h after liposome administration, whereas some larger LDs left the ER and were distributed in the cytoplasm 12 h after liposome administration (Fig. 5C, D). These data demonstrate that liposome-induced de novo LD biogenesis occurs on the ER membrane.

Physiological and pathological stressors disrupt ER homeostasis, and this is referred to as ER stress. For instance, abnormal lipid incorporation into the ER may contribute to the initiation of ER stress (51, 52). Accumulating evidence suggests that ER stress is one of the features of the foam cells that are found in atherosclerotic plaques (53, 54). ER expansion is a hallmark of ER stress (55). In this study, we also found that liposome uptake triggers ER expansion in M2 macrophages (Fig. 5B, D), indicating that LD development might induce ER stress. ER stress signaling occurs through the activation of three ER transmembrane sensors, and these signaling pathways activate inositol-requiring transmembrane kinase/endoribonuclease 1α (IRE1α)-mediated, activating transcription factor 6 (ATF6)-mediated, and protein kinase-like ER kinase (PERK)-mediated transcriptional programs (56). To verify this hypothesis, we evaluated ER homeostasis and stress before/after liposome administration. Using RT-qPCR, the markers of the ER stress signaling pathways were shown to be expressed at higher levels in macrophages after liposome uptake (Fig. 5E), indicating that the liposome treatment induced ER stress and activated ER sensor pathways in M2 macrophages.

Different lines of research have revealed that pathways activated by ER stress can initiate sterile inflammation (56). In response to ER stress, the IRE1α-mediated pathway initiates the degradation of IκB and thereby leads to NF-κB activation. Activated NF-κB migrates into the nucleus and induces the transcription of inflammatory genes, such as TNF-α, IL-1β, and IL-6 (57). Next, we investigated whether ER stress is involved in the reactivation of liposome-treated macrophages. BAY 11-7082 is an inhibitor of IκBα phosphorylation and NF-κB (58). After BAY 11-7082 treatment, the gene expression of proinflammatory cytokines in liposome-treated macrophages was significantly blocked (Fig. 5F). It becomes clear that liposome uptake triggers macrophage inflammatory activation via the ER stress-mediated IRE1α/NF-κB pathway.

### Dysfunctional BM niche macrophages affect hematopoiesis after systemic liposome delivery

**Fig. 6:**
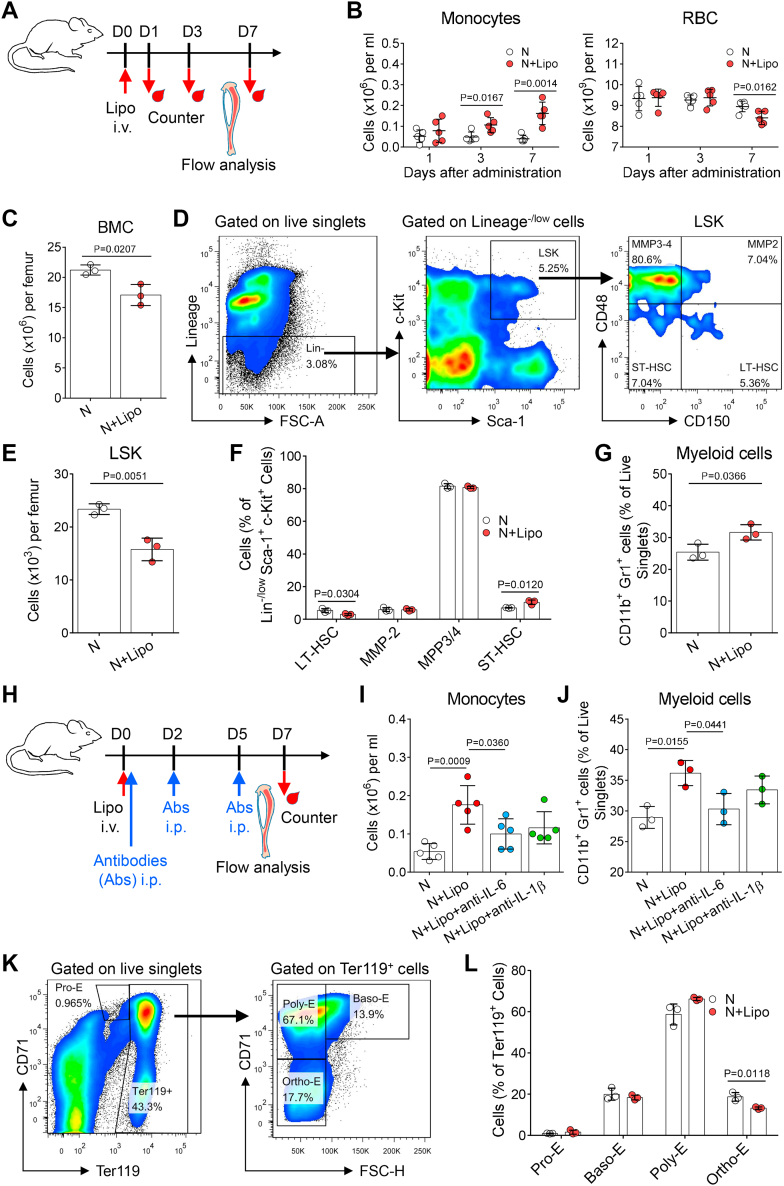
Dysfunctional BM niche macrophages affect hematopoiesis after liposome delivery. A: Experimental setup. Bones and peripheral blood samples were harvested for analysis at the indicated time points after liposome injection. B: Changes in peripheral blood monocyte and red blood cell counts in mice after liposome administration. The dots represent data from individual mice (n = 5). C: The number of BM nucleated cells in the femurs of mice 7 days after liposome administration. The dots represent data from individual mice (n = 3). D: Gating strategy for the quantification of LSK (Lin^−^c-Kit^+^Scal-1^+^) cells. E: The number of LSK cells in the femurs of mice 7 days after liposome administration. The dots represent data from individual mice (n = 3). F: The percentage of LT-HSCs, short-term HSCs, MPP2, and MPP3/4 cells in the LSK cell population. The dots represent data from individual mice (n = 3). G: The percentage of CD11b^+^Gr-1^+^ cells in the BM nucleated cell population. Dots are data from individual mice (n = 3). H: Experimental setup. The mice were injected with liposomes and then treated with anti-IL-6 blockade, anti-IL-1β blockade at the indicated time points, or no treatment (control). Then bones and peripheral blood samples were harvested for analysis. I: Changes in peripheral blood monocyte counts after liposome administration and antibody treatment. The dots represent data from individual mice (n = 5). J: The percentage of CD11b^+^Gr-1^+^ cells in the BM nucleated cell population after liposome administration and antibody treatment. The dots represent data from individual mice (n = 3). K: Gating strategy for the quantification of proerythroblasts (Pro-E), basophilic erythroblasts (Baso-E), polychromatic erythroblasts (Poly-E), and orthochromatic erythroblasts (Ortho-E) in flow cytometry plots of CD71 and Ter119 stained single cells. L: The percentage of Pro-E, Baso-E, Poly-E, and Ortho-E cells in BM Ter119^+^ cells of mice 7 days after liposome administration. The dots represent data from individual mice (n = 3). All experiments were repeated at least twice, and representative results are shown.

Next, we investigated the effect of liposome-treated macrophages on BM hematopoiesis (Fig. 6A). Changes in peripheral blood cell counts are indicative of HSC dynamics in the BM niche. Interestingly, we found that liposomes induced long-term changes in peripheral blood cell counts, such as an increased number of monocytes and a decreased number of red blood cells (Fig. 6B and supplemental Fig. S4A). These results indicate that there might be persistent perturbation of the BM HSC niche after systemic liposome administration.

To verify this hypothesis, we investigated the dynamics of BM cells on day 7 after liposome administration. A significant decrease in the total number of nucleated BM cells in liposome-treated mice was found (Fig. 6C). Next, we analyzed HSCs and hematopoietic progenitor cells (Lin^−^Sca1^+^c-Kit^+^; LSK) in the BM (Fig. 6D) (59). Compared with untreated mice, the percentage and absolute numbers of total LSK cells in liposome-treated mice were decreased (Fig. 6E and supplemental Fig. S4B). LSK cells can be further subdivided into long-term HSCs (LT-HSCs), short-term HSCs, MPP2 (multipotent hematopoietic progenitor cell), and MPP3/4 (Fig. 6D) (60). Mice treated with liposomes had a decreased percentage of LT-HSCs and an increased percentage of short-term HSCs in the LSK cell population (Fig. 6F). Moreover, the absolute numbers of total LT-HSCs in the BM of liposome-treated mice were significantly decreased (supplemental Fig. S4C), indicating that HSC homeostasis was disrupted after liposome delivery.

In addition to HSCs/hematopoietic progenitor cells, we also analyzed other blood cells in the BM. We found that the percentage of CD11b^+^Gr-1^+^ myeloid cells among nucleated BM cells was significantly increased after liposome administration (Fig. 6G). These data indicated that liposome administration induced myeloid-biased differentiation and output, which was consistent with the findings of the peripheral blood count (Fig. 6B). IL-6 and IL-1β have been shown to regulate myeloid differentiation (61, 62, 63, 64). Is the increased level of IL-6 and/or IL-1β in the extracellular fluid of the BM a factor that accounts for myeloid differentiation after liposome administration? To verify this hypothesis, mice were injected with liposomes and then treated with anti-IL-6 blockade, anti-IL-1β blockade, or no treatment (control) (Fig. 6H). Compared with the control group, the liposome-treated mice that received IL-6 blockade showed a reduced number of peripheral blood monocytes and percentage of BM myeloid cells, whereas these mice that received IL-1β blockade exhibited no overall changes (Fig. 6I, J), indicating that BM niche macrophage-derived IL-6 rather than IL-1β promoted myeloid differentiation in a paracrine manner after liposome administration.

To investigate the effect of liposome-induced BM macrophage dysfunctional on erythropoiesis, Ter119 and CD71 were used to map erythroblast development (Fig. 6K). Using flow cytometry, we found that liposome injection markedly reduced the percentage of orthochromatic erythroblasts among Ter119^+^ cells (Fig. 6L), indicating that the late erythroid maturation was blocked. A significant decline in the absolute number of orthochromatic erythroblasts was observed after liposome administration (supplemental Fig. S4D). The peripheral blood cell count results showed that liposome delivery reduced the number of mature red blood cells in bloodstream (Fig. 6B), which is consistent with the findings of BM analyses. Taken together, these data suggest that dysfunctional BM macrophages affect hematopoiesis after engulfing liposomes.

## Discussion

Liposomes have been widely studied for the delivery of different types of medicines, such as nucleic acids, molecular drugs, and diagnostic reagents. Fortunately, a few of these NP platforms have resulted in drug formulations that have reached clinical development (1). Although liposomes are engulfed by target cells after their systemic administration, most liposomes are cleared by macrophages in the MPS, such as the liver, spleen, BM, and lymph nodes (7). The macrophages of the MPS are also part of the innate immune system, which has raised the question of whether liposome uptake by phagocytic cells affects their functions. Multiple in vitro safety studies have been performed (65); however, there are very few studies that have focused on the toxic of drug carriers on macrophages in the MPS in vivo (66, 67).

A liposome is a spherical vesicle containing lipids, similar to the compositions of LDL. Macrophages engulf LDL and form foam cells in blood vessel walls, and their critical immune functions are diminished concurrently (68). We showed that liposome uptake induced LD formation in macrophages, giving them a foam cell-like appearance. This result is consistent with the findings by Fujita *et al.* (40, 41). Alterations in LD numbers and LD protein composition are proved to correlate with changes in immune cell function, such as myeloid cell activation (42). The present study revealed that LD formation was responsible for the inflammatory activation of macrophages after liposome uptake. Moreover, this study also revealed the central importance of LD biogenesis-induced ER stress in the inflammatory activation of liposome-treated macrophages.

Different from other MPS organs, the BM is the primary site of hematopoiesis. From another point of view, the BM is the “fertile soil” that nurses HSCs. BM niche macrophages play crucial roles in erythroid maturation and HSC maintenance at homeostasis (20, 21, 69). Dysfunction of BM niche macrophages results in abnormal hematopoiesis and various diseases (23, 24). In this study, we found that liposome administration elicited the proinflammatory activation of BM niche macrophages and affected hematopoiesis, such as by causing myeloid-biased output and impaired erythropoiesis. Our results are consistent with other reports showing that IL-6 and IL-1β are particularly important regulators of myeloid differentiation that function in a paracrine manner (61, 62, 63, 64). There might be several reasons that explain the impaired erythropoiesis. First, proinflammatory macrophages have been proven to be dysfunctional in iron recycling and phagocytosis (70). Moreover, IL-6 has been proven to limit the availability of iron to erythroblasts during stages of hemoglobin synthesis (71, 72). These findings arouse our attention to the application of lipid-based NPs in clinical practice, especially for patients with infection or BM hematopoietic dysfunction. Moreover, peripheral blood cell counts are an important index for clinical diagnosis. We observed persistent changes in peripheral blood cell counts after liposome administration, which must be taken in consideration when patients receive liposomal nanoformulations.

## Conclusion

Here, we investigated the potential adverse effects of liposomes on hematopoiesis. Liposome uptake induces macrophage “foam” cell formation and inflammatory activation in vitro and in vivo. We further provided evidence that liposome degradation after liposome engulfment causes LD formation and accumulation in macrophages, and LD biogenesis-mediated ER stress is involved in the inflammatory activation of macrophages. In addition, we also showed the effect of dysfunctional BM niche macrophages on hematopoiesis in mice. Altogether, this study draws attention to the safety of nanomedicines in clinical practice.

## Data availability

All data are included in the article.

## Supplemental data

This article contains supplemental data.

## Conflict of interest

The authors declare that they have no conflicts of interest with the contents of this article.
