# Supplementary material for: Liposomes trigger bone marrow niche macrophage “foam” cell formation and affect hematopoiesis in mice
Source: J Lipid Res. 2022 Sep 7;63(10):100273. doi: 10.1016/j.jlr.2022.100273 (PMC9587404; doi:10.1016/j.jlr.2022.100273)
Supplement: Supplemental Figures S1–S4 [file mmc1.docx]

Supplementary Materials

Liposomes trigger bone marrow niche macrophage “foam” cell formation and affect hematopoiesis in mice

Yue Li^1†^, Ran Yao^1†^, Miao Ren^2,3,4^, Ke Yuan^2,3,4^, Yuwei Du^2,3,4^, Yuan He^5^, Haiquan Kang^1,6^, Shengnan Yuan^1^, Wen Ju^2,3,4^, Jianlin Qiao^2,3,4^, Kailin Xu^2,3,4^, Lingyu Zeng^2,3,4*^

^1^School of Medical Technology, Xuzhou Medical University, Xuzhou, Jiangsu, China

^2^Blood Diseases Institute, Xuzhou Medical University, Xuzhou, Jiangsu, China

^3^Key Laboratory of Bone Marrow Stem Cell, Xuzhou, Jiangsu, China

^4^Department of Hematology, the Affiliated Hospital of Xuzhou Medical University, Xuzhou, Jiangsu, China

^5^School of Pharmacy, Xuzhou Medical University, Xuzhou, Jiangsu, China

^6^Department of Laboratory Medicine, the Affiliated Hospital of Xuzhou Medical University, Xuzhou, Jiangsu, China

^†^These authors have contributed equally to this work and share first authorship

***Corresponding author:**

Lingyu Zeng, Email: [zengly2000@163.com](mailto:zengly2000@163.com).

**Supplementary figures 1-4**

**
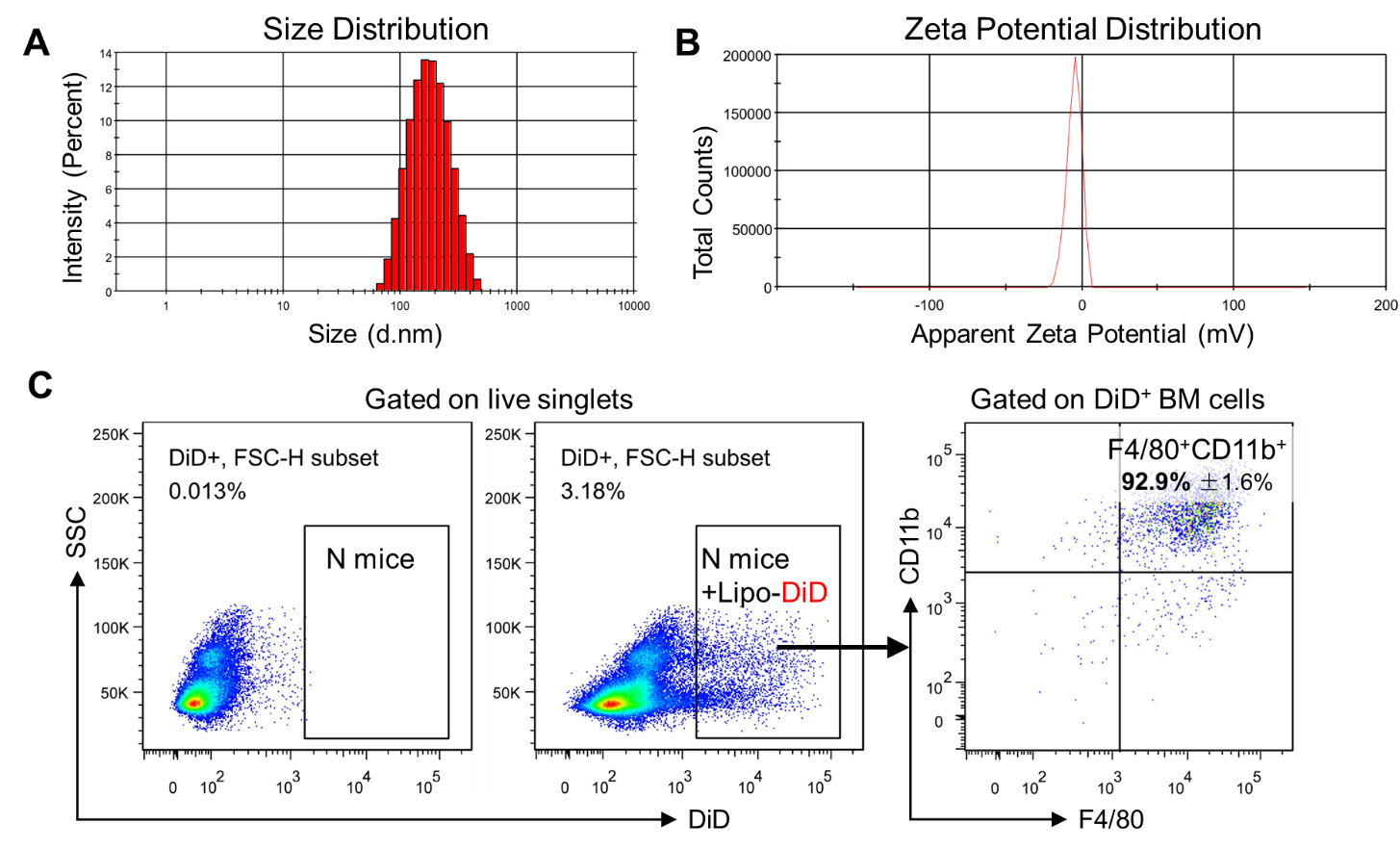
**

**Supplementary Figure 1. Characterization of liposomes *in vitro* and *in vivo*.** **(A)** The size distribution of liposomes. **(B)** The zeta potential distribution of liposomes. **(C)** Flow cytometer was used to identify the cell type of DiD^+^ BM cells in mice after DiD-loaded liposome injection. The data were calculated from individual mice (n=3). All experiments were repeated at least twice, and representative results are shown.

**
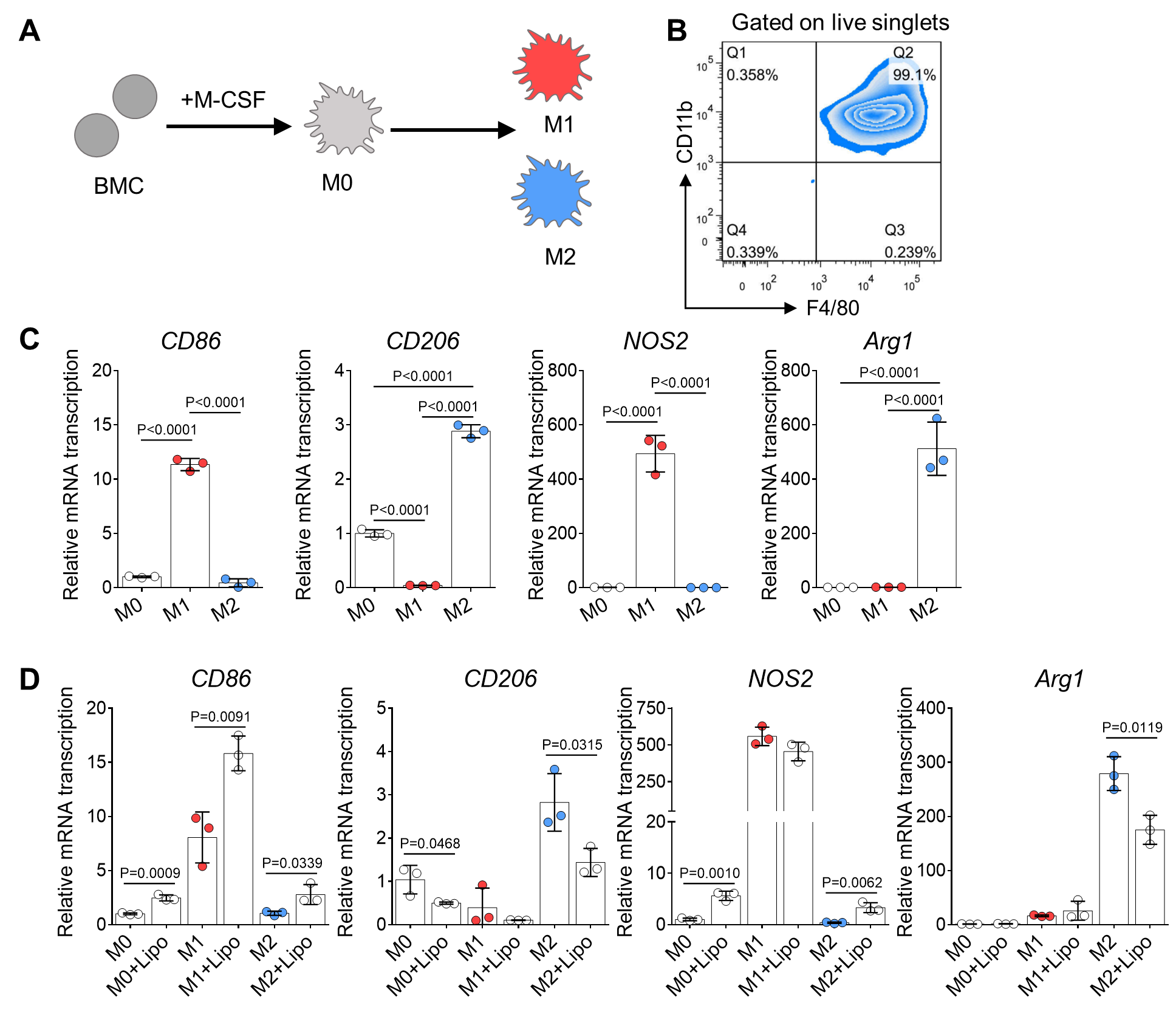
**

**Supplementary Figure 2.** **Effect of liposome uptake on macrophage activation.** **(A)** Schematic diagram showing the culture and activation of primary macrophages. **(B)** After 5 days of M-CSF treatment, flow cytometer was used to assess the purity of *in vitro-*cultured macrophages (F4/80 and CD11b). **(C)** 24 h after cytokine stimulation, RT-qPCR was used to assess *CD86*, *CD206*, *NOS2* and *Arg-1* gene expression in activated macrophages. One dot represents the data from macrophages derived from one mouse (n=3). **(D)** 12 h after liposome administration, RT-qPCR was used to assess *CD86, CD206, NOS2* and *Arg1* gene expression in macrophages. One dot represents the data from macrophages derived from one mouse (n=3). All experiments were repeated at least twice, and representative results are shown.


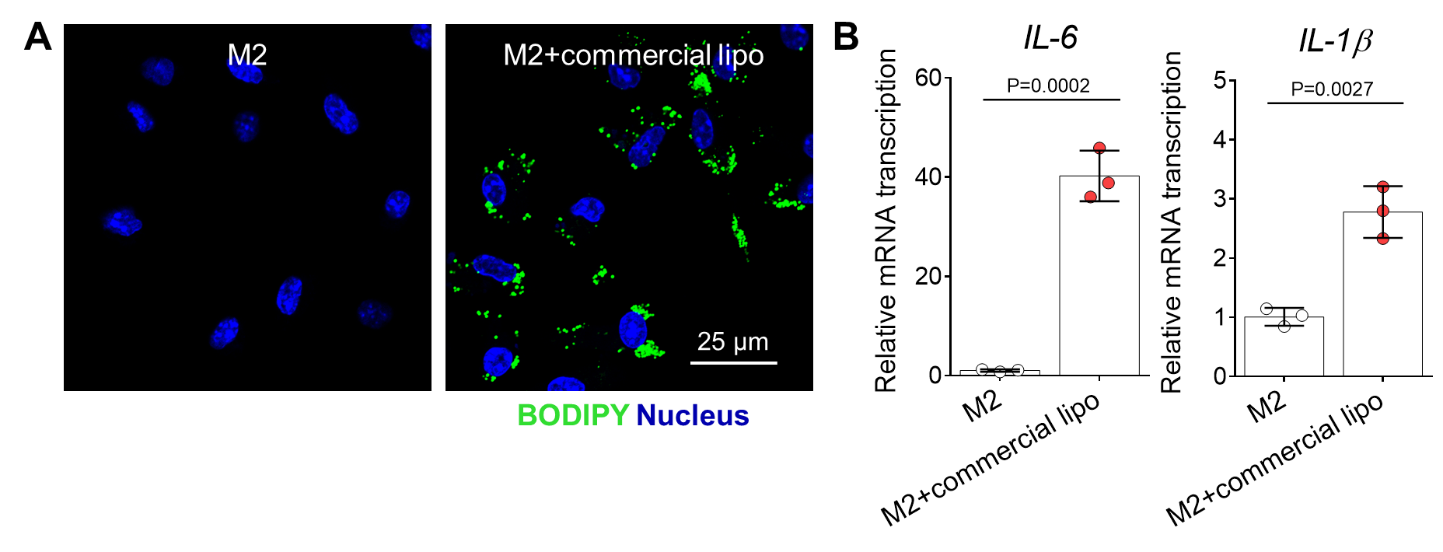


**Supplementary Figure 3.** **Effect of commercial liposomes on macrophage LD accumulation and functions.** **(A)** Representative fluorescence confocal microscopy images of LDs (green color, BODIPY) and nuclei (blue color, Hoechst 33342) in M2 macrophages after commercial liposome administration. Scale bar, 25 µm. **(B)** 12 h after commercial liposome administration, RT-qPCR was used to assess *IL-6* and *IL-1β* gene expression in macrophages. One dot represents the data from macrophages derived from one mouse (n=3). All experiments were repeated at least twice, and representative results are shown.

**
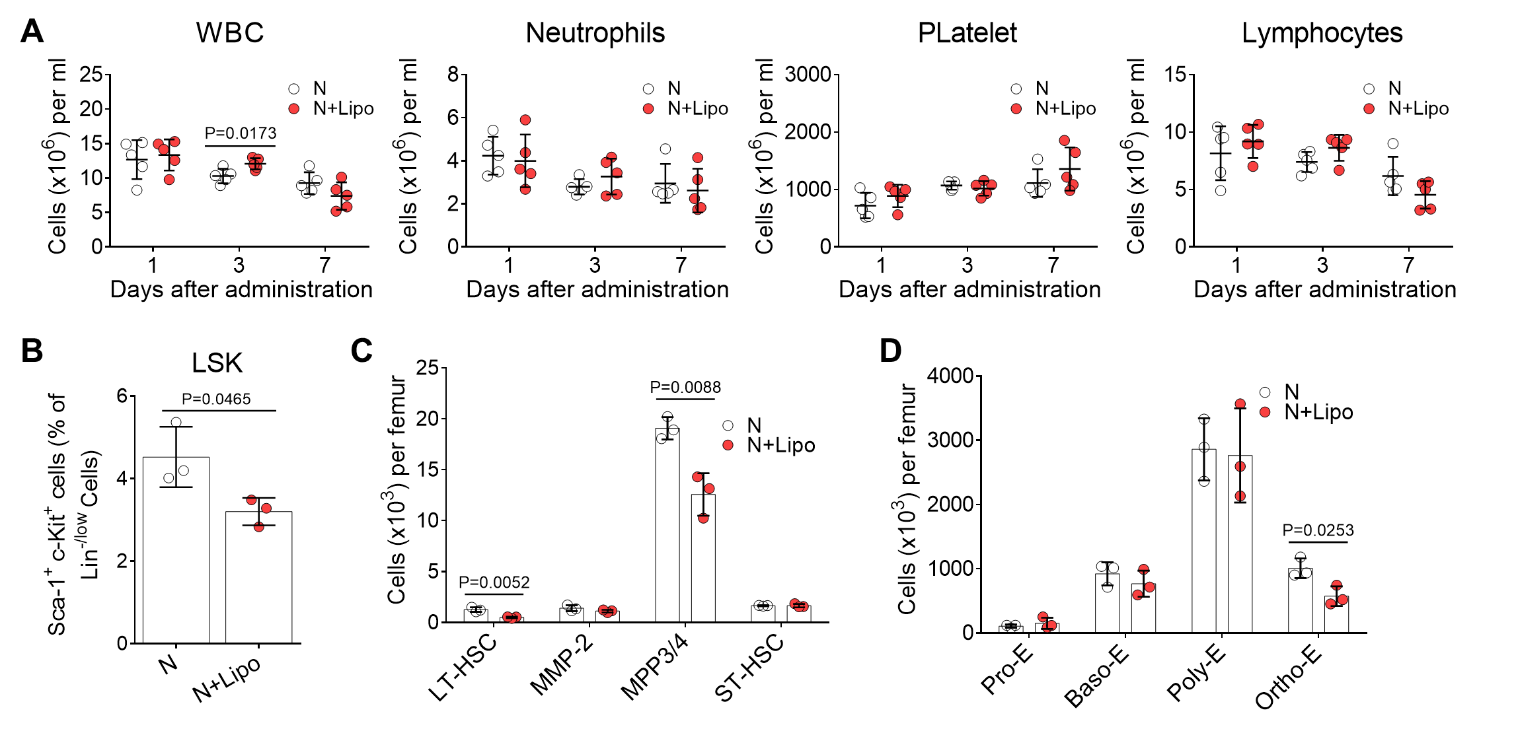
**

**Supplementary Figure 4. Effect of systemic liposome injection on peripheral blood and bone marrow hematopoiesis in mice.** **(A)** Changes in WBC, neutrophil, PLT and lymphocyte counts in the peripheral blood of mice after liposome administration. The dots represent data from individual mice (n=5). **(B)** The percentage of LSK in the BM nucleated cell population of mice 7 d after liposome administration. The dots represent data from individual mice (n=3). **(C)** The absolute number of long-term HSCs (LT-HSCs), short-term HSCs (ST-HSCs), MPP2, and MPP3/4 cells in the BM of mice 7 d after liposome administration. The dots represent data from individual mice (n=3). **(D)** The absolute number of proerythroblasts (Pro-E), basophilic erythroblasts (Baso-E), polychromatic erythroblasts (Poly-E) and orthochromatic erythroblasts (Orto-E) in the BM of mice 7 d after liposome administration. The dots represent data from individual mice (n=3). All experiments were repeated at least twice, and representative results are shown.
